# Naloxone Accessibility Under the State Standing Order Across Mississippi

**DOI:** 10.1001/jamanetworkopen.2023.21939

**Published:** 2023-07-06

**Authors:** Emily Gravlee, Sujith Ramachandran, Anne Cafer, Erin Holmes, Jacob McGregor, Taylor Jordan, Meagen Rosenthal

**Affiliations:** 1 Department of Pharmacy Administration, The University of Mississippi School of Pharmacy, University; 2 Center for Pharmaceutical Marketing and Management, The University of Mississippi School of Pharmacy, University; 3 Department of Sociology and Anthropology, The University of Mississippi, University; 4 Department of Pharmacy Practice, The University of Mississippi School of Pharmacy, University; 5 now with Mississippi Baptist Medical Center, Jackson

## Abstract

**Importance:**

Naloxone is a life-saving medication for individuals experiencing an opioid overdose. Naloxone standing orders aim to make naloxone more available by allowing patients improved access to this medication at community pharmacies; however, lawful availability does not mean that this life-saving intervention is accessible to patients.

**Objective:**

To characterize naloxone availability and out-of-pocket cost under the state standing order in Mississippi.

**Design, Setting, and Participants:**

This telephone-based, mystery-shopper census survey study included Mississippi community pharmacies open to the general public in Mississippi at the time of data collection. Community pharmacies were identified using the Hayes Directories April 2022 complete Mississippi pharmacy database. Data were collected from February to August 2022.

**Exposures:**

Mississippi House bill 996, the Naloxone Standing Order Act, signed into law in 2017, allowing pharmacists to dispense naloxone under a physician state standing order at a patient’s request.

**Main Outcomes and Measures:**

The main outcomes were naloxone availability under Mississippi’s state standing order and the out-of-pocket cost of available formulations.

**Results:**

There were 591 open-door community pharmacies surveyed for this study, with a 100% response rate. The most common pharmacy type was independent (328 [55.50%]), followed by chain (147 [24.87%]) and grocery store (116 [19.63%]). When asked, “Do you have naloxone that I can pick up today?” 216 Mississippi pharmacies (36.55%) had naloxone available for purchase under the state standing order. Of the 591 pharmacies, 242 (40.95%) were unwilling to dispense naloxone under the state standing order. Among the 216 pharmacies with naloxone available, the median out-of-pocket cost for naloxone nasal spray (n = 202) across Mississippi was $100.00 (range, $38.11-$229.39; mean [SD], $105.58 [$35.42]) and the median out-of-pocket cost of naloxone injection (n = 14) was $37.70 (range, $17.00-$208.96; mean [SD], $66.62 [$69.27]).

**Conclusions and Relevance:**

In this survey study of open-door Mississippi community pharmacies, availability of naloxone was limited despite standing order implementation. This finding has important implications for the effectiveness of the legislation in preventing opioid overdose deaths in this region. Further studies are needed to understand pharmacists’ unwillingness to dispense naloxone and the implications of lack of availability and unwillingness for further naloxone access interventions.

## Key Points

**Question:**

What is the accessibility of naloxone under the state standing order implemented in 2017 in Mississippi?

**Findings:**

In this survey study of 591 Mississippi community pharmacies, only 37% had naloxone immediately available under the state standing order and 41% were unwilling to dispense naloxone under the state standing order.

**Meaning:**

The findings suggest that poor naloxone availability may pose a challenge to both state and federal interventions aiming to improve naloxone access.

## Teaser

This survey study evaluates the availability and out-of-pocket costs of naloxone among community pharmacies under a state standing order in Mississippi.

## Introduction

Naloxone is a prescription opioid antagonist that is available as a nasal spray or injection and is indicated for reversal of opioid overdose.^1^ Naloxone has been identified as a life-saving medication for individuals with opioid use disorder,^2^ with multiple health stakeholders, including the Centers for Disease Control and Prevention^3^ and the US Food and Drug Administration (FDA),^4^ encouraging action to increase uptake. In the face of the opioid epidemic, naloxone access has become especially pertinent. In an effort to combat opioid-related deaths, many states have expanded access to naloxone using state standing orders. Naloxone standing orders authorize pharmacists to supply naloxone to patients under a standing prescription, which eliminates the need for a patient-prescriber encounter to authorize the medication. The first standing order for naloxone was created in 2007, and sharp adoption of these laws occurred in 2013.^5^ To date, 47 states have express standing orders for naloxone.^6^ Existing evidence from the National Bureau of Economic Research indicates that among states that have enacted these measures, a 9% to 11% decrease in opioid-related deaths has been observed.^6^ However, naloxone may not be accessible to patients despite it being lawfully attainable without a prescription.

Naloxone availability at community pharmacies has been noted to vary widely based on geographic location.^7,8,9,10^ Nationally, previous studies found that statewide or regionwide naloxone availability at pharmacies ranged from 30% to 95%.^8,9,11,12^ However, even if naloxone is available via standing orders and is in stock at many pharmacies,^6^ it is often not free. Naloxone costs, which are less frequently reported than availability, also vary widely. A study conducted in Philadelphia, Pennsylvania, found that out-of-pocket (OOP) costs for naloxone nasal spray ranged from $119 to $150.^8^ However, naloxone costs in California ranged from $45 to $260 for intranasal naloxone formulations and from $27 to $4500 for injectable formulations.^12^ Despite the number of studies performed to assess naloxone availability and cost, representation of rural, southern regions in the literature is lacking. Importantly, the South may be where observed gaps in naloxone accessibility are most magnified. Mississippi has the lowest median annual household income among US states,^13^ and almost 40% percent of all Mississippians are Black individuals.^14^ Understanding naloxone availability and costs in this state is paramount in ensuring sociodemographically equitable access to this life-saving medication. Additionally, examination of these factors within Mississippi may provide limited insight into naloxone access issues in the southern US more broadly.

To improve naloxone access across the state, Mississippi House bill 996, the Naloxone Standing Order Act, was signed into law in 2017 and allows pharmacists to dispense the opioid antagonist naloxone under a standing blanket prescription at a patient’s request.^15^ However, even after 5 years of state endorsement, to our knowledge, no studies have explored the effectiveness of the law for making naloxone accessible to Mississippians. The importance of understanding naloxone availability in the context of pharmacy willingness to stock and dispense the medication has been magnified by recent events. In early 2023, the FDA approved a naloxone nasal spray (Narcan) for over-the-counter use,^16^ and in December 2022, the Mississippi State Department of Health announced that naloxone would be free to Mississippians via a waiver program that allows individuals to purchase naloxone at the pharmacy with no OOP cost or to have a naloxone kit provided to them via the mail free of charge.^17^ However, even these interventions to improve access may prove to be unsatisfactory if naloxone is not physically present in Mississippi communities. From January 2020 to January 2022, opioid-related drug overdose deaths in Mississippi spiked by 122%.^18^ Ensuring that policies intended to increase naloxone access are effective is vital in saving Mississippians’ lives.^19^ This study aimed to characterize the effectiveness of the Mississippi state standing order for naloxone by evaluating naloxone availability and OOP cost under the state standing order. Ultimately, naloxone’s accessibility under the state standing order may prove to be telling for success of further access interventions in the state and broadly across the South.

## Methods

**Figure 1.**
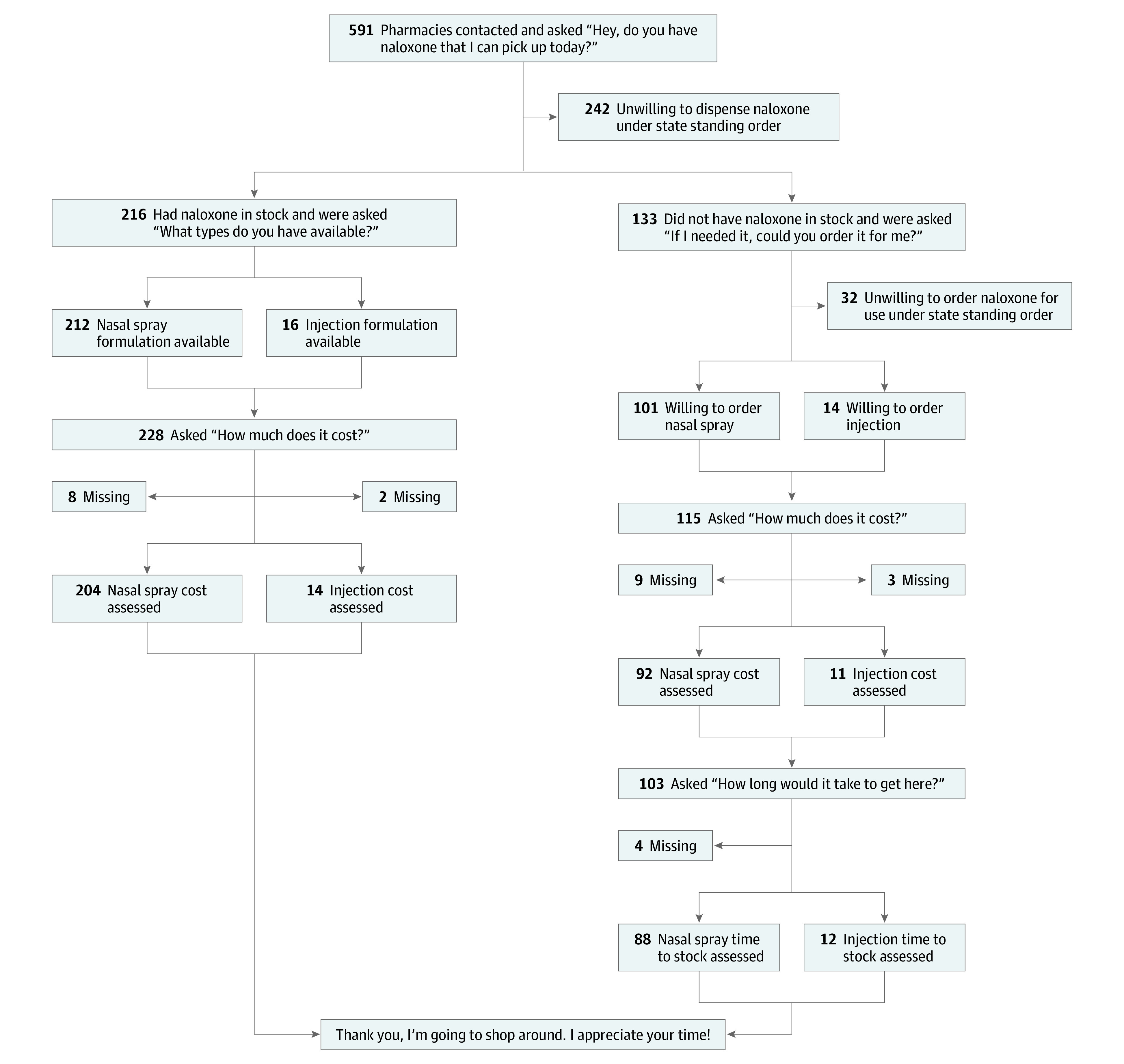
Survey Items, Structure, and Attrition

This study followed the American Association for Public Opinion Research (AAPOR) reporting guideline.^20^ This was a telephone-based, mystery-shopper census survey study of Mississippi community pharmacies. Notably, a census design indicates that 100% of the sampling frame was attempted to be reached in the study. Data were collected by 22 volunteers from February to August 2022. Most volunteers (21 [95.45%]) were recruited from The University of Mississippi School of Pharmacy student body, which is largely composed of in-state residents. The majority of volunteer callers (19 [86.36%]) were female. Volunteers called pharmacies to collect survey data according to the survey flow in Figure 1. If asked by a pharmacy staff member, volunteers were without a prescription for naloxone and were uninsured. Responses to the survey were recorded by project volunteers in Qualtrics survey software.^21^ This study was not considered human subjects research by The University of Mississippi institutional review board and was exempt from review. Informed consent was not obtained because the study was not considered human participant research.

### Data Sources

This study used a cross-sectional census design in which all retail pharmacies in Mississippi were surveyed. A list of pharmacies was obtained from the Hayes Directories April 2022 complete Mississippi pharmacy database.^22^ The Hayes Directories data sets contain pharmacy information from state boards of pharmacy and include retail pharmacies open to the general public. In addition to exclusion of pharmacies in hospital or ambulatory care settings imposed by the data set, pharmacies were excluded from the study if they were housed inside a subscription-based wholesale store. Sampling frame and study eligibility were determined prior to the study start, and in accordance with AAPOR guidance, the sampling frame completeness was evaluated.^20^ To verify Hayes Directories data set completeness, a manual completeness check of a random 10% sample of counties was performed using Google searches. In this search, no discrepancies were identified between the manual search and the Hayes Directories data set, resulting in identification of 100% of community pharmacies in Mississippi at the time of the study. Pharmacy type was identified by the student volunteer as a grocery store, chain, or independent pharmacy.

### Measures

In accordance with AAPOR guidelines, measures used within the survey are available in Figure 1.^20^ Similar to previous studies, availability^10,11^ and OOP cost^8^ of naloxone formulations, including both branded and generic injectable and nasal spray formulations, were assessed. Availability was a dichotomous variable based on same-day availability at the time of the pharmacy call. Out-of-pocket cost was measured in US dollars. Naloxone availability under the state standing order was assessed by pharmacies’ response to the initial question, “Do you have naloxone that I can pick up today?” (Figure 1). If pharmacies indicated that naloxone was not immediately available, naloxone was determined to be unavailable. Pharmacy willingness to dispense naloxone was indirectly assessed based on information provided by the pharmacy during the course of the call. For example, if the pharmacy indicated that any additional action was required to obtain naloxone, including a prescription, naloxone was noted to be unavailable and the pharmacy was listed as unwilling to dispense naloxone under the state standing order. Pharmacies that did not object to providing naloxone to the mystery caller were noted as willing to dispense naloxone under the state standing order. If a pharmacy had no available naloxone but was willing to dispense naloxone under the state standing order, willingness to order naloxone was assessed using the question, “If I needed it, could you order it for me?” (Figure 1). If a pharmacy indicated that it was willing to order naloxone for the caller, the time to stock naloxone in hours and OOP cost of the medication were gathered.

### Statistical Analysis

Naloxone availability, willingness to dispense, OOP cost, willingness to order, and time to stock were descriptively analyzed using SAS, version 5.2 (SAS Institute Inc). Naloxone availability and OOP cost of naloxone nasal spray were mapped at a county level using R, version 12.0 (R Project for Statistical Computing). Additionally, QGIS, version 3.30 (QGIS Association) (eMethods in Supplement 1) was used to generate maps examining naloxone availability by county according to the proportion of the county that consisted of racial and ethnic minority populations (eFigure 1 in Supplement 1), number of opioid overdose deaths in 2020 (eFigure 2 in Supplement 1), and proportion of the county living below the poverty line (eFigure 3 in Supplement 1). These additional maps were generated to provide insight into sociodemographic disparities in naloxone access under the state standing order. The decision to dichotomously map racial and ethnic minority populations vs White populations was driven by the racial makeup of the state and our desire to concisely convey visual information regarding naloxone access by racial or ethnic makeup.

## Results

### Availability and OOP Cost

There were 591 open-door community pharmacies identified in Mississippi. Data were collected from each pharmacy, resulting in a 100% response rate for the study. The most common pharmacy type was independent (328 [55.50%]), followed by chain (147 [24.87%]) and grocery store (116 [19.63%]). Of the 82 Mississippi counties, 81 (98.78%) had a pharmacy present in the county, of which almost one-fifth (16 [19.75%]) had no naloxone access under the state standing order. Of the 591 pharmacies, 216 (36.55%) had naloxone immediately available for purchase under the state standing order; 242 pharmacies surveyed (40.95%) were unwilling to dispense naloxone under the state standing order. Overall, just over half of pharmacies (317 [53.64%]) had naloxone available immediately or were willing to order it for purchase under the state standing order. Table 1 provides a breakdown of naloxone availability by pharmacy type. Independent pharmacies had the lowest proportion of naloxone available (84 of 328 [25.61%]), while grocery store pharmacies had the highest proportion of naloxone available (65 of 116 [56.03%]). The most commonly stocked formulation of naloxone was the nasal spray (212 [98.15%]). Only 16 pharmacies (7.41%) in the state stocked the injection formulation, while 12 pharmacies (5.56%) stocked both the injection and the nasal spray formulations. The median OOP cost for naloxone nasal spray was $100.00 (range, $38.11-$229.39; mean [SD], $105.58 [$35.42]), and the median OOP cost for the naloxone injection was $37.70 (range, $17.00-$208.96; mean [SD], $66.62 [$69.27]). These prices were compared with the anticipated average wholesale price of naloxone to be dispensed under Mississippi’s state standing order using unit pricing estimates from Lexicomp (Table 2) and assuming a 20% markup for point of sale at the pharmacy. These pricing estimates ranged from $155 to $180.

**Table 1.** Naloxone Availability Under the Mississippi State Standing Order by Pharmacy Type

Pharmacy type	Pharmacies with naloxone available for same-day pickup, No./total No. (%)
Independent	84/328 (25.61)
Chain	67/147 (45.58)
Grocery store	65/116 (56.03)

**Table 2.** Naloxone Formulations Available Under the Mississippi State Standing Order by Unit Price and Anticipated Sales Price Estimates

Approved product formulations	Quantity	Unit price, $^a^	Total anticipated sales price, $^b^
Naloxone prefilled syringe	2 Syringes	18.81-19.80	45.14-47.52
Naloxone, 0.4 mg/mL, single-dose vial	2 Vials	5.27-23.72	12.65-56.93
Naloxone prefilled syringe (Zimhi)	1 Case	75.00	90.00
Naloxone prefilled syringe (Zimhi)	1 Carton	75.00	180.00
Naloxone nasal spray (Narcan)	2-Pack kit	75.00	180.00
Naloxone nasal spray	2-Pack kit	64.80-71.25	155.52-171.00
Naloxone nasal spray (Kloxxado)	2-Pack kit	75.00	180.00

^a^ Pricing information was obtained from Lexicomp.

^b^ Total unit price per quantity with a markup of 20% added.

**Figure 2.**
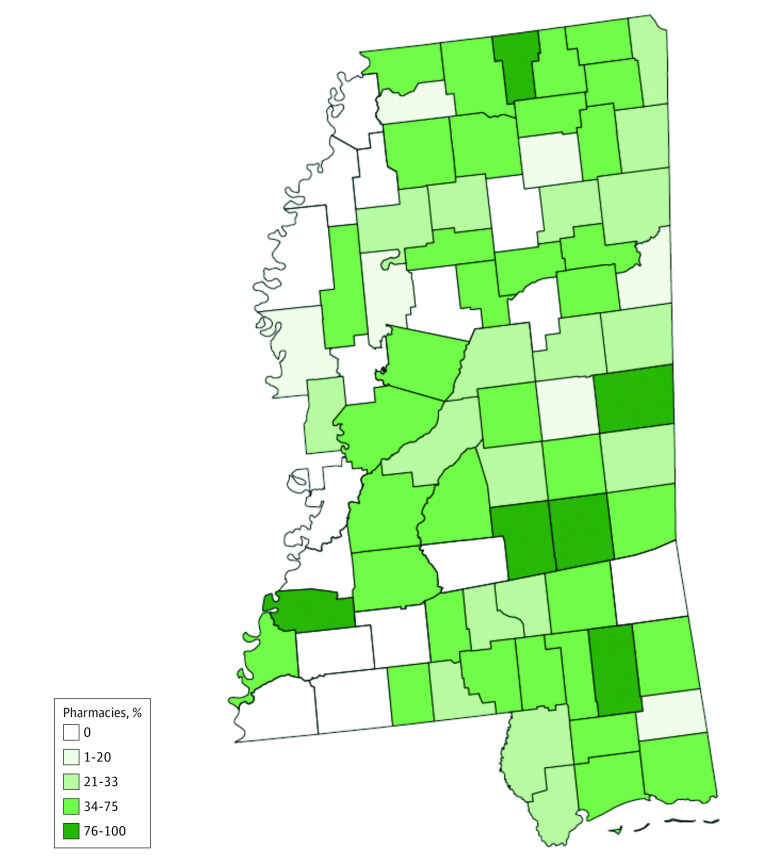
Naloxone Same-Day Availability Under a Standing Order in Mississippi by County

**Figure 3.**
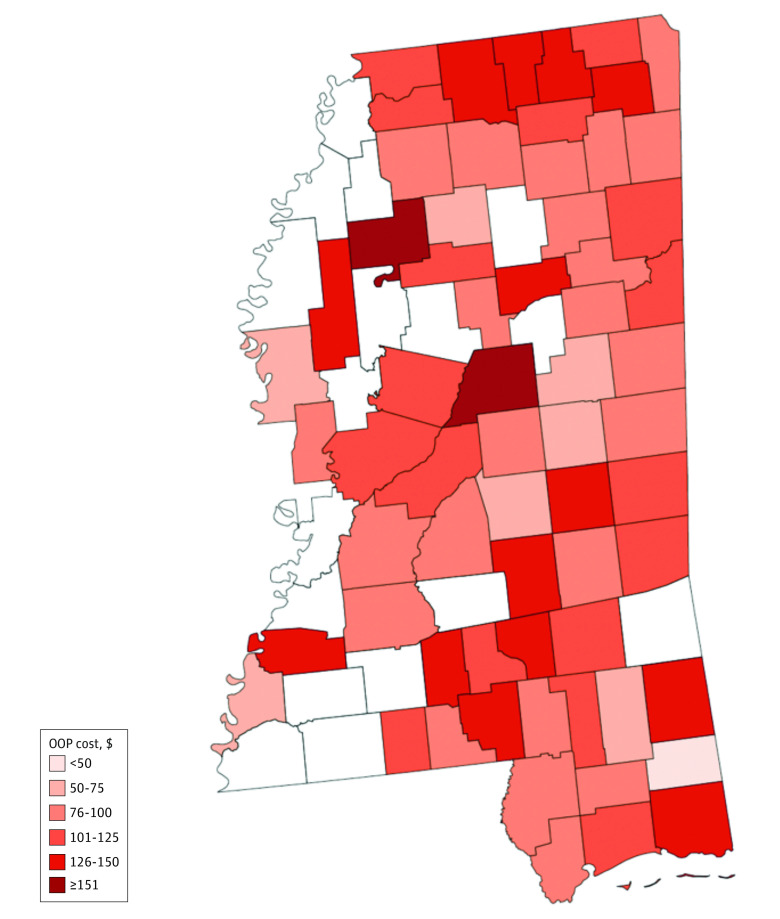
Naloxone Nasal Spray Cash Out-of-Pocket (OOP) Cost in Mississippi by County White counties represent areas with no information available on naloxone nasal spray cost.

Naloxone availability was mapped at the county level (Figure 2). Unavailability of naloxone was concentrated along the western border encompassing the Delta region of the state, which is recognized for its long-standing income^23^ and health^24^ disparities. Because of the limited number of pharmacies reporting stock of injectable naloxone (n = 16), only naloxone nasal spray OOP cost was mapped (Figure 3). Mapping naloxone cost across the state revealed that quoted OOP costs for naloxone were higher across the northern border of the state near Tennessee. Naloxone nasal spray OOP costs were quoted to be higher than $150 in both Attala and Tallahatchie counties. Almost half (30 of 64 [46.88%]) of all counties where a pharmacy stocked naloxone nasal spray had it available for $100 or less.

### Willingness to Order and Time to Stock

Among 133 pharmacies without available naloxone but willing to dispense it under the standing order, 101 (75.94%) were willing to order naloxone. For these pharmacies, the median time to stock naloxone nasal spray (n = 88) was 24 hours (range, 6-336 hours; mean [SD], 41.04 [38.89] hours), and the median time to stock naloxone injection formulations (n = 12) was 24 hours (range 24-72 hours; mean [SD], 28.00 [13.86] hours).

## Discussion

While the standing order for naloxone exists across Mississippi, this study showed that less than half of all Mississippi pharmacies had naloxone immediately available for consumers under the state standing order. The proportion of pharmacies with naloxone available in this census was lower than most others found in literature^25^ and notably even lower than what was found in a study examining naloxone access across community pharmacies within Alabama,^11^ which is a socioeconomically comparable state. Notably, independent pharmacies were the pharmacy type with the lowest proportion of naloxone available, which is consistent with other literature.^10,12^ This finding may be driven by the fact that, while chain pharmacies often implement organizational policies to encourage naloxone stocking,^26,27^ independent pharmacies face no such pressure or incentive to stock naloxone. When we examined access to naloxone at a county level rather than aggregating to the total number of pharmacies in the state, we found that almost one-fifth (16 of 81 [19.75%]) of Mississippi counties containing pharmacies had no naloxone access under the state standing order. Furthermore, a high proportion of pharmacies indicated that they were unwilling to dispense naloxone under the state standing order. These findings suggest that standing order legislation alone was not sufficient to improve naloxone access for Mississippians and that the ability of this state-level public health policy to address overdose deaths was limited.

Quoted prices and price ranges for naloxone found in this study align with existing literature, which has demonstrated wide variability in pricing strategies.^8,12^ Alignment with literature, however, does not mean that these quoted prices should not be probed. Given that the average wholesale price for a naloxone nasal spray for sale, as directed by the standing order (Table 2), ranged from $155 to $180, higher-end OOP cost estimates provided by pharmacies exceeded the anticipated cost to consumer of this drug under the state standing order.^28^ For example, 1 pharmacy’s quoted cost of $229.39 represented a markup of roughly 27% for a branded naloxone nasal spray package or 34% to 48% for a generic package (Table 2). Because pricing varied substantially at the point of sale, these costs may demonstrate pricing choices made by pharmacies. To compound this issue, to our knowledge, no studies have evaluated price sensitivity of naloxone for patients. While 1 study identified that naloxone was price inelastic, the study did not specifically examine price elasticity for uninsured individuals^29^ and contradicted more recent evidence suggesting that price sensitivity is a barrier to naloxone use, especially for those who are uninsured.^30^ In the context of over-the-counter naloxone, lack of appropriate pricing strategies suited to over-the-counter sales may prohibit naloxone use. Further studies focusing on identifying naloxone price sensitivity for patients across Mississippi and more broadly across the US should be conducted to guide an appropriate over-the-counter pricing strategy for this medication. Without interventions to ensure affordability, naloxone may be unattainable for many Mississippians.

However, Mississippi continues to aim to improve naloxone access for its residents. In December 2022, the Mississippi State Department of Health implemented a program to eliminate naloxone costs to Mississippians either by mail kits provided through the Department of Health or by payment waivers used at the point of purchase at a pharmacy.^17,31^ Payment waivers can remove cost barriers to naloxone for Mississippians but do not address the lack of naloxone availability that this study observed at community pharmacies across the state. The Mississippi State Department of Health’s naloxone mail kit option intends to overcome both cost barriers and gaps in naloxone availability at community pharmacies but faces shortcomings due to awareness, time to delivery, and gaps in Mississippi’s internet infrastructure. Naloxone access is contingent on access to internet services in a state where roughly one-fifth of households lack internet services.^32^ While mail-order naloxone is better than no naloxone, mail-order options may prevent patient interaction with pharmacists who can provide critical counseling and administration information about this life-saving medication. Ultimately, failure to address community pharmacy availability of naloxone may hinder the uptake of naloxone for those in need.

In addition to Mississippi-driven interventions to improve access, federal movements exist for improving naloxone accessibility. In late March 2023, the FDA approved a naloxone nasal spray (Narcan) for over-the-counter use.^16^ With this change, naloxone standing orders may become obsolete. Ideally, like ibuprofen, naloxone will be available at every pharmacy. Similarly, without a prescription, consumers may be required to assume full OOP costs of these products. Assuming that the Mississippi naloxone OOP cost waiver stands, action by the FDA has the potential to greatly increase naloxone accessibility across the state. However, if the waiver is removed or altered, over-the-counter access to naloxone may prove to be cost prohibitive for many Mississippians, as Mississippi has the nation’s highest percentage of people living in poverty^33^ and 1 of the highest rates of uninsured patients.^19^ Most importantly, reasons for low naloxone availability observed in this study are not understood. Previous literature has identified that many factors, including pharmacists’ attitudes toward naloxone, influence naloxone availability, but few of these studies examining pharmacist attitudes have been carried out in the southeastern US.^11,34^ If we fail to understand and address why poor naloxone uptake exists across community pharmacies in Mississippi, even the best-designed interventions may fail to improve access. Therefore, studies that focus on pharmacists’ perceptions of over-the-counter naloxone across Mississippi, as well as willingness to stock over-the-counter naloxone, are warranted and may best serve to guide policy makers in improving naloxone access.

### Strengths and Limitations

This study has strengths. It provides insight into naloxone availability under a state standing order in the general population. The telephone-based, mystery-shopper census survey study design ensured an adequate response rate and minimized response bias for pharmacies. Most importantly, this study emulated the patient experience in attempting to access naloxone under the state standing order across Mississippi.

This study also has limitations. When capturing naloxone OOP costs, investigators were unable to assess whether the price of branded or generic naloxone was being quoted. This made analysis of availability for brand vs generic naloxone formulations impractical. Additionally, due to unfamiliarity, pharmacy staff members may have felt uncomfortable answering the mystery caller. While this ideally should not have influenced report of naloxone availability, this may have biased collected naloxone availability. Furthermore, *naloxone* sounds like *suboxone*, which is commonly used for medication-assisted treatment of opioid use disorder and is a federally regulated Schedule III medication.^35^ Pharmacy staff may have been hesitant to discuss naloxone over the telephone if they believed that mystery callers were inquiring about a scheduled medication. To mitigate this, if the pharmacy staff member answering the telephone asked the mystery caller to repeat what medication they were requesting, the mystery caller would respond, “Naloxone, like Narcan.” Narcan is a widely recognized brand name for naloxone that is distinct from branded names of suboxone.^35^ However, whether this strategy improved recognition of the request for naloxone is unknown, and lack of clear understanding about the call’s subject may have downwardly biased naloxone availability found in this study. In addition, other types of unmeasured errors intrinsic to survey-type research may have influenced the observed findings.^20^

## Conclusions

In this survey study of naloxone availability under the state standing order across Mississippi community pharmacies, despite lawful availability, naloxone was not widely accessible to patients. A total of 40.95% of all pharmacies surveyed were unwilling to dispense naloxone under the state standing order, demonstrating that despite legislative action, the standing order has not been widely adopted by pharmacy stake holders. Findings from this study also have implications for recent interventions to improve access to naloxone in the state. Mississippi State Department of Health and FDA interventions aim to make naloxone affordable and available over the counter for consumers. However, given the low standing order availability and high unwillingness to dispense naloxone observed, questions about pharmacy adoption and appropriate implementation of the over-the-counter rollout remain.

## Notes

Supplement 1. **eMethods.** Description of Data Sources and Mapping Procedures for Additional Maps **eFigure 1.** Naloxone Same-Day Availability Under Standing Order in Mississippi by County and Proportion of Mississippi County Consisting of Racial and Ethnic Minority Populations **eFigure 2.** Naloxone Same-day Availability Under Standing Order in Mississippi by County and Number of Opioid Overdose Deaths per County in 2020 **eFigure 3.** Naloxone Same-Day Availability Under Standing Order in Mississippi by County and Proportion of the County Living Below the Poverty Line in 2020
eReferences

Supplement 2.
Data Sharing Statement
