# Supplementary material for: Naloxone Accessibility Under the State Standing Order Across Mississippi
Source: JAMA Netw Open. 2023 Jul 6;6(7):e2321939. doi: 10.1001/jamanetworkopen.2023.21939 (PMC10326645; doi:10.1001/jamanetworkopen.2023.21939)
Supplement: Supplement 1. — eMethods. Description of Data Sources and Mapping Procedures for Additional Maps eFigure 1. Naloxone Same-Day Availability Under Standing Order in Mississippi by County and Proportion of Mississippi County Consisting of Racial and Ethnic Minority Populations eFigure 2. Naloxone Same-day Availability Under Standing Order in Mississippi by County and Number of Opioid Overdose Deaths per County in 2020 eFigure 3. Naloxone Same-Day Availability Under Standing Order in Mississippi by County and Proportion of the County Living Below the Poverty Line in 2020 eReferences [file jamanetwopen-e2321939-s001.pdf]

Supplemental Online Content

Gravlee E, Ramachandran S, Cafer A, et al. Naloxone accessibility under the state standing order across Mississippi. *JAMA Netw Open*. 2023;6(7):e2321939. doi:10.1001/jamanetworkopen.2023.21939

eMethods. Description of Data Sources and Mapping Procedures for Additional Maps
eFigure 1. Naloxone Same-Day Availability Under Standing Order in Mississippi by County and Proportion of Mississippi County Consisting of Racial and Ethnic Minority Populations
eFigure 2. Naloxone Same-Day Availability Under Standing Order in Mississippi by County and Number of Opioid Overdose Deaths per County in 2020
eFigure 3. Naloxone Same-Day Availability Under Standing Order in Mississippi by County and Proportion of the County Living Below the Poverty Line in 2020
eReferences

This supplemental material has been provided by the authors to give readers additional information about their work.

© 2023 Gravlee E et al. *JAMA Network Open*.

eMethods. Description of Data Sources and Mapping Procedures for Additional Maps

Datasets and Mapping Procedures

In addition to mapping naloxone access broadly across the state, naloxone same-day availability under state standing order was mapped according to three county-level factors: racial makeup of the county; proportion of households in the county under the poverty line; and number overdose deaths in the county occurring in 2020. County-level racial makeup was assessed as proportion of the county consisting of racial and ethnic minority populations. Proportion of households under the poverty line was assessed using American Community Survey 2020 data.[1] Data for racial makeup of counties was gathered from 2020 Mississippi Census data.[1] Notably, due to issues in data collection, these estimates are vintage estimates and do not necessarily represent actual Census counts for year 2020.[2] Overdose deaths at the county-level were assessed using the MS Opioid and Heroin Data Collaborative's Provisional Data Report Calendar Year 2020.[3] All mapping was performed in QGIS v3.30.

© 2023 Gravlee E et al. *JAMA Network Open*.

eFigure 1. Naloxone Same-Day Availability Under Standing Order in Mississippi by County and Proportion of Mississippi County Consisting of Racial and Ethnic Minority Populations

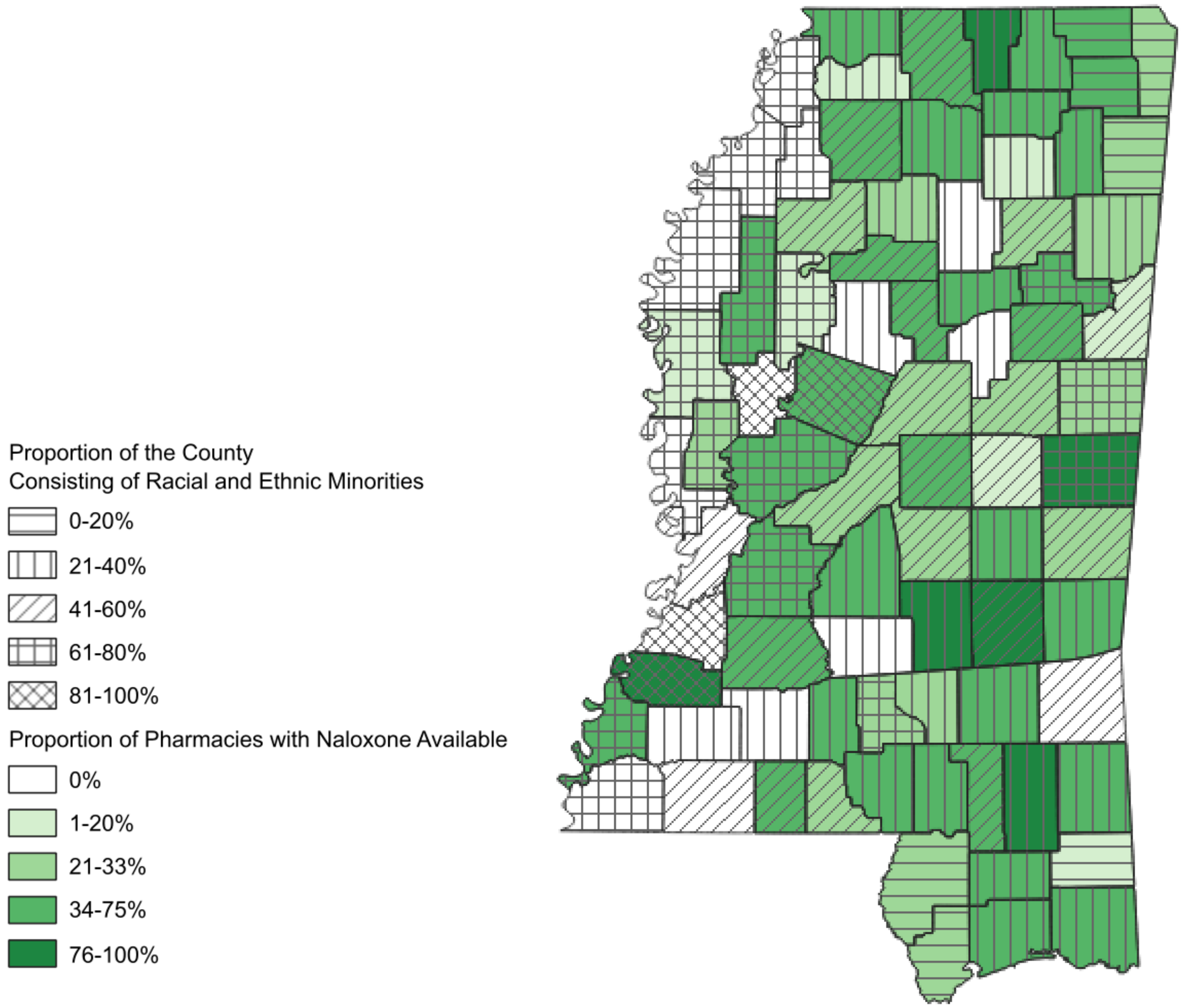

© 2023 Gravlee E et al. *JAMA Network Open*.

eFigure 2. Naloxone Same-Day Availability Under Standing Order in Mississippi by County and Number of Opioid Overdose Deaths per County in 2020

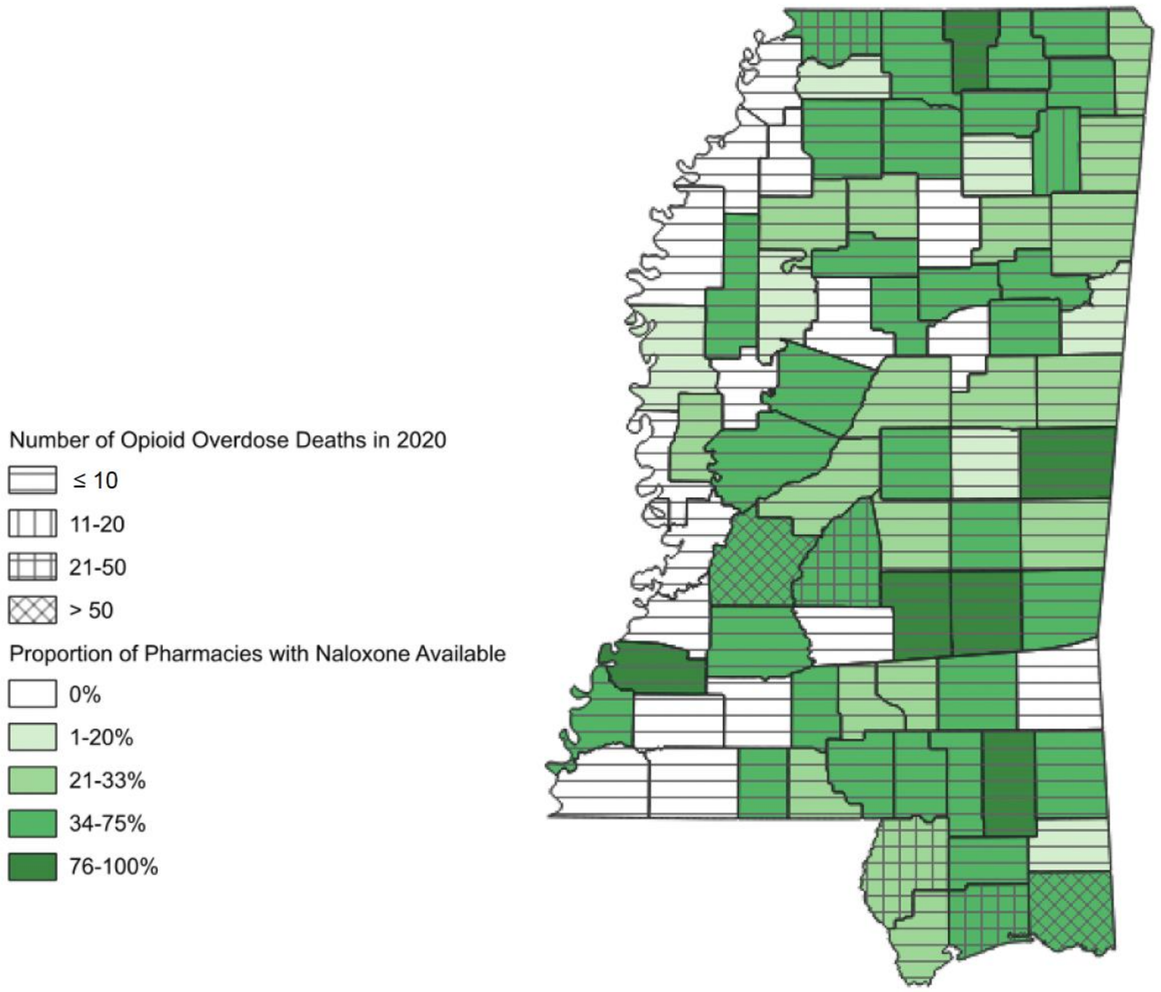

© 2023 Gravlee E et al. *JAMA Network Open*.

eFigure 3. Naloxone Same-Day Availability Under Standing Order in Mississippi by County and Proportion of the County Living Below the Poverty Line in 2020

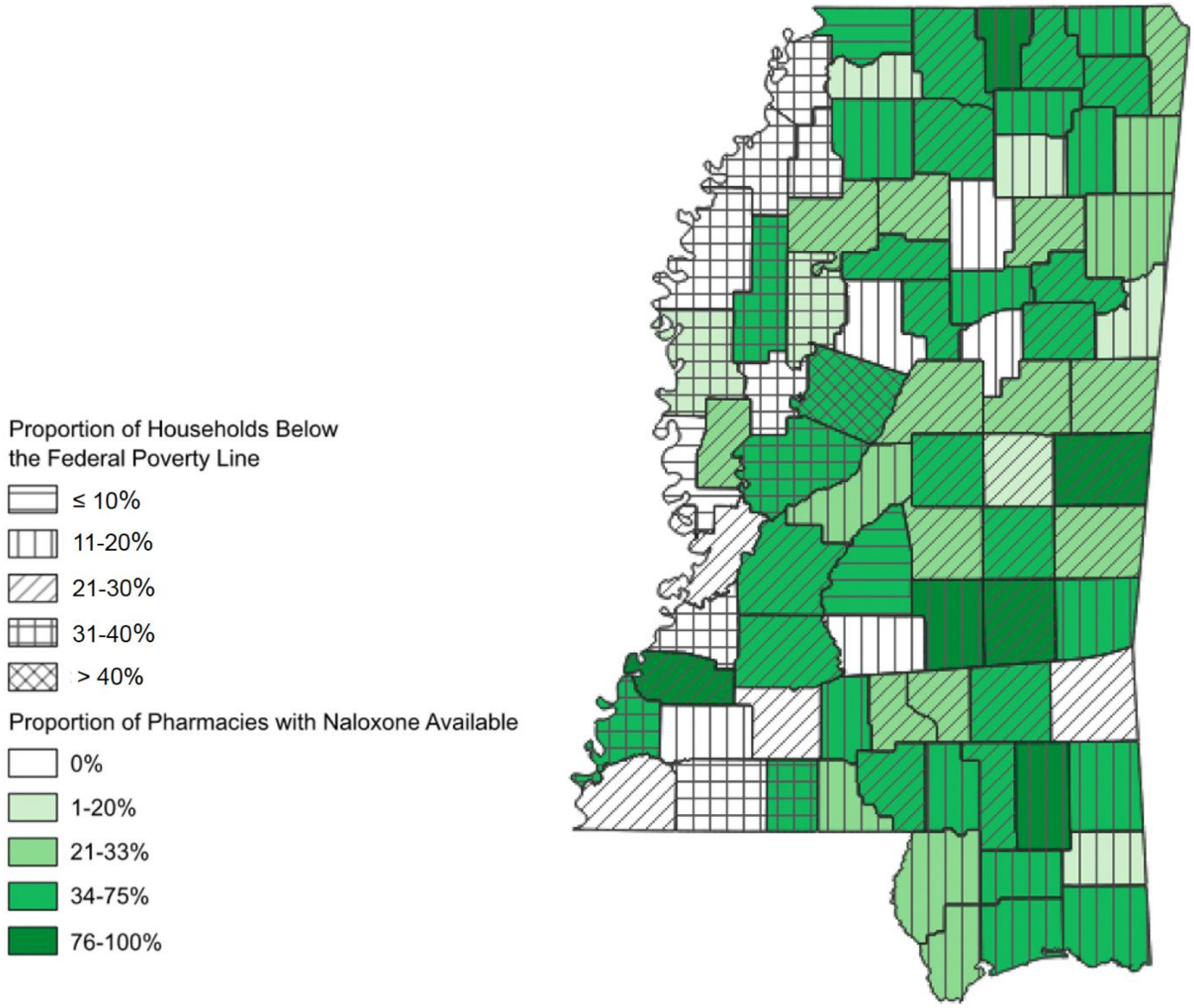

© 2023 Gravlee E et al. *JAMA Network Open*.

eReferences

1. American Community Survey (ACS). Census.gov. Accessed February 13, 2023. https://www.census.gov/programs-surveys/acs.

2. Next 2020 Census Data Products to be Released in 2023. Census.gov. Accessed September 20, 2022. https://www.census.gov/newsroom/press-releases/2022/2020-census-data-products-schedule-2023.html.

3. Provisional Data Report Calendar Year 2020. The Mississippi Opioid and Heroin Data Collaborative. Published April 11, 2021. https://www.msdh.ms.gov/msdhsite/_static/resources/13236.pdf.

© 2023 Gravlee E et al. *JAMA Network Open*.
