# Supplementary material for: Naloxone Accessibility Under the State Standing Order Across Mississippi
Source: JAMA Netw Open. 2023 Jul 6;6(7):e2321939. doi: 10.1001/jamanetworkopen.2023.21939 (PMC10326645; doi:10.1001/jamanetworkopen.2023.21939)
Supplement: Supplement 2. — Data Sharing Statement [file jamanetwopen-e2321939-s002.pdf]

Data Sharing Statement

Gravlee. Naloxone Accessibility Under the State Standing Order Across Mississippi. *JAMA Netw Open*. Published July 06, 2023. doi:10.1001/jamanetworkopen.2023.21939

Data
Data available: No

Additional Information
Explanation for why data not available: Data for this manuscript could potentially be used to identify pharmacies.
